# Blood Level of Glial Fibrillary Acidic Protein (GFAP) Does not Correlate With Disease Progression in a Rat Model of Familial ALS (SOD1^G93A^ Transgenic)

**DOI:** 10.3389/fneur.2018.00954

**Published:** 2018-11-14

**Authors:** Jeremy Jeffrey, Hannah D'Cunha, Masatoshi Suzuki

**Affiliations:** ^1^Department of Comparative Biosciences, University of Wisconsin-Madison, Madison, WI, United States; ^2^The Stem Cell and Regenerative Medicine Center, University of Wisconsin-Madison, Madison, WI, United States

**Keywords:** glial fibrillary acidic protein (GFAP), amyotrophic lateral sclerosis, a rat model of familial ALS, ELISA, Biomarkers

## Abstract

Amyotrophic lateral sclerosis (ALS) is a neurodegenerative disease characterized by specific loss of motor neurons in the spinal cord and brain stem. Currently, there are limited options for treating ALS and further investigation of the disease etiology and ALS disease progression need to be completed. There is an urgent need to identify biomarkers to detect and study disease progression in ALS. Glial fibrillary acidic protein (GFAP) is an intermediate filament protein that is expressed by a number of cells related to the central nervous system including glial cells and ependymal cells. Recent studies indicated that significant levels of GFAP protein were detected in peripheral tissues, such as skeletal muscle. In this study, we hypothesized that levels of GFAP in blood represent a biomarker of disease progression in ALS. To test this specific hypothesis, we used a rat model of familial ALS (SOD1^G93A^ transgenic), which has been extensively used to understand the complexity of this devastating disease. Disease progression in a cohort of male and female SOD1^G93A^ transgenic rats was monitored by motor function, and blood samples were collected when these animals reached disease end-stage. We measured GFAP protein levels by ELISA and found no correlation between GFAP concentration and disease progression in either serum and plasma samples of SOD1^G93A^ transgenic. Further investigation would be required in order to implicate blood GFAP as a potential biomarker for ALS.

## Introduction

Amyotrophic lateral sclerosis (ALS) is a debilitating neuromuscular disease caused by the loss of motor neurons in the brain and spinal cord (1, 2). Approximately, 90% of all ALS patients are considered as sporadic while the remaining 10% are caused by a familial/hereditary genetic etiology. There are a number of genes in which ALS-causing mutations have been characterized, such as *SOD1, TARDPB, FUS*, and *C9ORF72* (2). While the exact mechanism of pathology remains unknown, multiple pathologies have been involved in ALS; abnormal protein misfolding and aggregation (3), axonal transport defects, glutamate excitotoxicity (4), oxidative stress (5), mitochondrial dysfunction (6), neuroinflammation (7, 8), and astrocyte activation (9, 10). To date, there are limited options for treating ALS and further investigation of the disease etiology and ALS disease progression need to be completed. Identification of novel biomarkers has the potential of greatly contributing necessary information that could aid in understanding the disease progression of ALS.

In recent studies, glial fibrillary acidic protein (GFAP) has been proposed as a potential biomarker in patients with specific disease conditions associated with acute and chronic neurological diseases (11–15). GFAP is an intermediate filament protein that is expressed by a number of cells related to the central nervous system including glial cells and ependymal cells (16). Particularly, the expression of GFAP is commonly used as a marker for a subgroup of astrocytes (17). Increased levels of GFAP protein has been identified in the blood samples of patients suffering from neurological diseases, such as Parkinson's disease (13), intracerebral hemorrhage (14, 18), traumatic spinal cord injury (19), and multiple sclerosis (15). GFAP levels were also high in serum samples from the patients that exhibit motor and sensory neurological pathologies (11). In the case of ALS, GFAP has been shown to be increased in the cerebrospinal fluid from patients (20). This is reasonable based on the fact that astrocyte activation is one of the critical hallmarks that occur during the process of motor neuron degeneration in ALS (21, 22).

GFAP expression is not limited to the central nervous system and also found in the peripheral nervous system in specific neurological diseases including ALS (23, 24). Furthermore, we recently reported that an overall increase of GFAP expression following disease progression was determined in the limb muscle of a rat model of familial ALS (SOD1^G93A^ transgenic) (24). All together, these observations led us to hypothesize that blood GFAP concentration would be influenced by disease conditions in ALS. To test this hypothesis, we measured blood GFAP concentration in endpoint SOD1^G93A^ transgenic rats by ELISA (enzyme-linked immunosorbent assay).

## Materials and methods

### SOD1^G93A^ transgenic rats

We used a transgenic rat model that over-expresses a mutant form of superoxide dismutase 1 (SOD1) (25–27). SOD1^G93A^ transgenic male rats were bred with wild-type female Sprague Dawley rats in order to maintain the colony. Both strains of rats were obtained from Taconic (Hudson, NY). Heterozygous SOD1^G93A^ progeny were identified with polymerase chain reaction (PCR) using tail DNA with primers specific for the human SOD1 gene. They were maintained in a room with controlled illumination (lights on 0500–1900 h) and temperature (23 ± 1°C), and given free access to laboratory chow and tap water. The animal experiments in this study were performed in accordance with the animal care guidelines and regulations from the University of Wisconsin-Madison and National Institutes of Health.

### Blood sample collection

Serum and plasma samples were collected from end-stage SOD1^G93A^ transgenic rats. Humane end-stage was determined by using at least one of two criteria; the animal is unable to right itself within 30 seconds and/or a 20% loss of maximum body weight is reached (26, 28). Age-matched wildtype littermates were used as non-transgenic controls. For plasma preparation, whole blood was collected in blood collection tubes (EDTA-plasma tubes). In regards to serum preparation, the clotted blood was centrifuged and serum was carefully separated.

### ELISA (enzyme-linked immunosorbent assay)

GFAP protein levels were determined using a GFAP ELISA Kit (NS830, Millipore, Temecula, CA) while following the manufacturer's instructions. The samples and standard controls were applied to the 96-well plate pre-coated with anti-GFAP antibody (capture antibody) and incubated at room temperature for 2 hours on an orbital shaker. After incubation, the wells were washed with the washing buffer, biotinylated ant-GFAP detection antibody was added, and then the wells were incubated at room temperature for 1 h. The wells were then washed and incubated with streptavidin conjugated horseradish peroxidase for 30 min. After washing the wells, immobilized antibody-enzyme complexes were quantified by monitoring horseradish peroxidase activity in the presence of the substrate 3,3′,5,5′-tetramethylbenzidine. The absorbance of each sample and standard was read at 450 nm using a Chromate 4300 microplate reader (Awareness Technology, Palm City, FL).

### Statistical analysis

The GraphPad Prism software (La Jolla, CA, USA) was used for statistical analysis. The data was presented as mean ± SEM. Unpaired two-tailed Student's *t*-test was performed to compare two groups. Differences were considered significant when P < 0.05.

## Results

**Figure 1:**
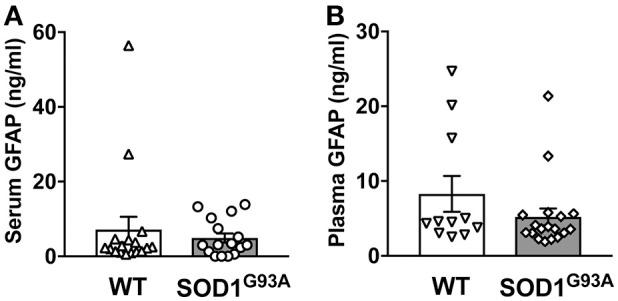
Blood GFAP concentration in SOD1^G93A^ transgenic and wild-type rats. A cohort of SOD1^G93A^ transgenic rats was monitored for disease progression until end-stage. Serum **(A)** and plasma **(B)** samples were then collected from SOD1^G93A^ transgenic rats and age-matched non-transgenic (wild-type, WT) littermates. GFAP protein levels were measured by ELISA.

**Figure 2:**
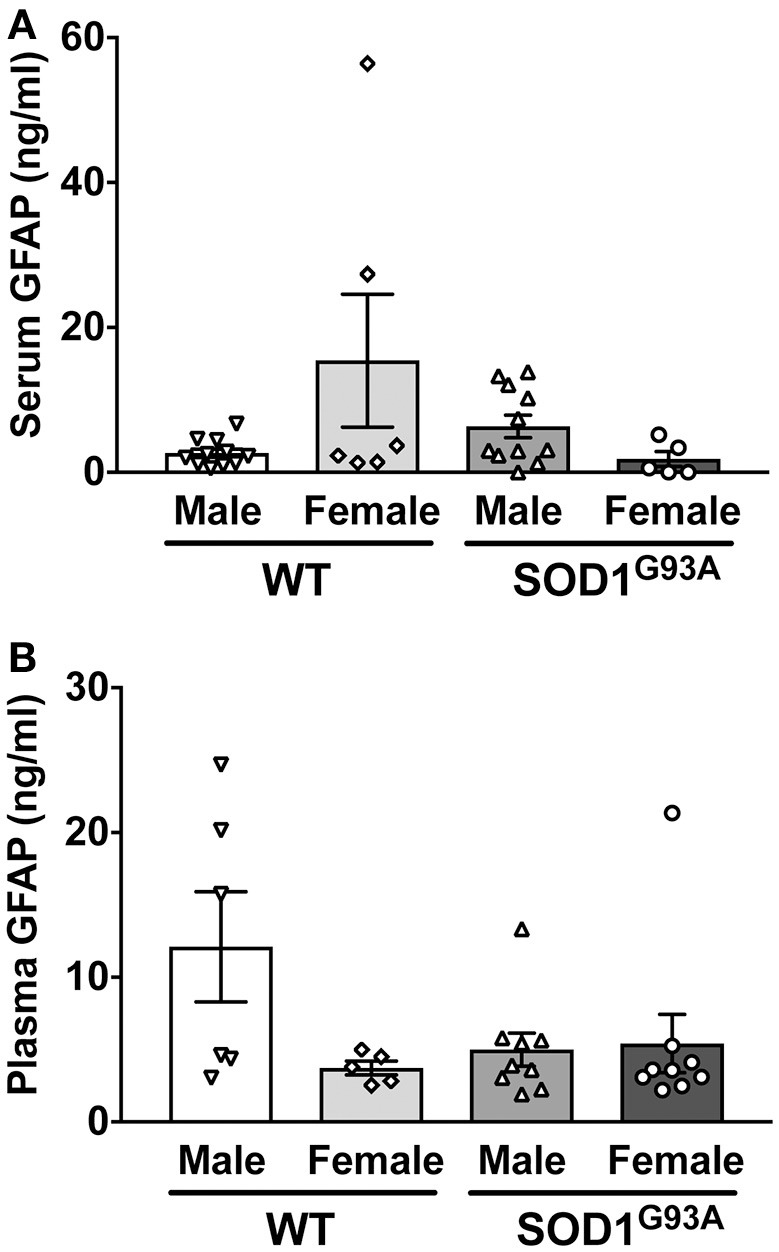
No sexual difference in serum **(A)** and plasma **(B)** GFAP levels in wild-type (WT) control and SOD1^G93A^ transgenic rats.

In order to determine serum GFAP levels in ALS model rats, 16 SOD1^G93A^ transgenic rats (11 males and 5 females) were compared to 17 age-matched wild-type littermate controls (11 males and 6 females). GFAP measurement by ELISA revealed that there was no significant difference in serum GFAP concentrations (Figure 1A). As we previously found sexual dimorphism in disease progression in SOD1^G93A^ transgenic rats (25, 26), we compared GFAP levels in male and female rats. There was no significant difference in serum GFAP concentration when we compared sexes in wild-type and SOD1^G93A^ rats (Figure 2A). It should be noted, however, that we observed high GFAP levels in two wild-type females (Figure 2A). Further, there was a trend of increased serum GFAP levels in SOD1^G93A^ males, although the difference did not reach statistical significance (*P* = 0.07). In this experiment, the survival period of female SOD1^G93A^ rats (175 ± 8.0 days of age, median = 176 days) was longer than male SOD1^G93A^ rats (168 ± 5.1 days of age, median = 162 days), which was consistent with our previous observations (25, 26).

Similarly, we measured plasma GFAP levels in another cohort of SOD1^G93A^ rats. There was no difference in plasma GFAP concentrations between SOD1^G93A^ transgenic rats (5.2 ± 1.1 ng/ml, *n* = 18) and wild-type controls (8.2 ± 2.4 ng/ml, *n* = 11; Figure 1B). Further, there was no sex difference in the plasma samples from SOD1^G93A^ transgenic rats (5.0 ± 1.1 ng/ml in male, *n* = 9; 5.4 ± 2.0 ng/ml in female, *n* = 9) and wild-type controls (9.6 ± 3.5 ng/ml in male, *n* = 6; 3.7 ± 0.5 ng/ml in female, *n* = 5; Figure 2B). Together, we conclude that there is no correlation between blood GFAP levels and disease progression in ALS.

## Discussion

Being that there are limited effective therapies for ALS, it is imperative to investigate the mechanisms that control disease progression. Identifying potential biomarkers will aid in the further understanding of these mechanisms during ALS pathology. As ALS is a late-onset and progressive disease, a new development of objective biomarkers is critical for early diagnosis or therapeutic monitoring (29). For instance, cerebrospinal fluid (CSF) levels of specific molecules, such as neurofilament proteins and Cystatin C, have been proposed as possible biomarkers in ALS. Interestingly, a recent study examined GFAP concentration in a small cohort of ALS patients, suggesting that GFAP might serve as a potential biomarker (20).

As we already described in the Introduction above, a number of previous studies support that GFAP is correlated to specific conditions in patients with various neurodegenerative diseases (11–15, 18, 19, 30). Additionally, GFAP levels considerably increased in CSF from patients suffering from Alexander disease (30), a genetic disorder caused by mutations in the GFAP gene, leading to myelin abnormalities in the midbrain and cerebellum (31). These studies support the idea that GFAP could be classified as a diagnostic biomarker in patients with neurological disease conditions.

The specific hypothesis that drove this pilot study was based on our recent study showing high levels of GFAP in peripheral tissues of ALS model rats (24). We compared GFAP levels in the skeletal muscle of SOD1^G93A^ transgenic rats at different time points; pre-symptomatic, symptomatic, and end-stage. We found that GFAP levels were elevated steadily from pre-symptomatic to endpoint stage. GFAP proteins were localized around the pre-synaptic regions in the beginning stages of disease. GFAP expression encompassed acetylcholine receptor-positive endplates at the neuromuscular junctions and around motor axons at end-stage. GFAP levels increased in end-stage ALS rats along with the activation of inflammatory cells, such as macrophages, this suggested that inflammation may be a responsible factor for the increase of active glial cells in the skeletal muscle of SOD1^G93A^ transgenic rats. Following these observations, we tested a specific hypothesis that increased levels of GFAP in skeletal muscles would largely influence protein concentration in ALS model rats. Although we were not aware of an obvious relationship between blood GFAP levels and disease progression in SOD1^G93A^ rats, we should acknowledge potential limitations of the current study. First, the mutation in our ALS model rats only represents a small population of ALS patients (1, 2). Therefore, a possibility still remains that different levels of blood GFAP may be identified in the other animal models of familial ALS with TARDPB, FUS, or C9ORF72 gene mutation. Given that only endpoint animals were used to represent severe disease conditions, additional studies would need to be conducted to determine GFAP levels at different timepoints of disease stage.

While we identified detectable levels of GFAP proteins in blood samples, it is unknown what type of cells and tissues largely contribute to sustain GFAP levels in the blood flow. Interestingly, some rats showed high levels of GFAP in both serum and plasma samples. There seems to be no specific trend in sex to have such variations in GFAP concentration. Additionally, no obvious correlation was identified between GFAP level and survival period. Further studies need to address whether these variations of GFAP concentration may represent specific physiological or disease conditions in rats.

## Author contributions

JJ and MS: conception and design. JJ and HD: performing experiments and collection and/or assembly of data. JJ and MS: data analysis and interpretation. JJ, HD, and MS: manuscript writing and final approval of manuscript.

### Conflict of interest statement

The authors declare that the research was conducted in the absence of any commercial or financial relationships that could be construed as a potential conflict of interest.

## References

[1] Peters OM Ghasemi M Brown Jr RH Emerging mechanisms of molecular pathology in ALS. J Clin Invest. (2015) 125:1767–79. 10.1172/JCI71601 25932674 PMC4463186

[2] Ajroud-Driss S Siddique T. Sporadic and hereditary amyotrophic lateral sclerosis (ALS). Biochim Biophys Acta (2015) 1852:679–84. 10.1016/j.bbadis.2014.08.010 25193032

[3] Blokhuis AM Groen EJ Koppers M van den Berg LH Pasterkamp RJ. Protein aggregation in amyotrophic lateral sclerosis. Acta Neuropathol. (2013) 125:777–94. 10.1007/s00401-013-1125-6 23673820 PMC3661910

[4] Bogaert E d'Ydewalle C Van Den Bosch L. Amyotrophic lateral sclerosis and excitotoxicity: from pathological mechanism to therapeutic target. CNS Neurol Disord Drug Targets (2010) 9:297–304. 10.2174/187152710791292576 20406181

[5] Barber SC Shaw PJ. Oxidative stress in ALS: key role in motor neuron injury and therapeutic target. Free Radic Biol Med. (2010) 48:629–41. 10.1016/j.freeradbiomed.2009.11.018 19969067

[6] Duffy LM Chapman AL Shaw PJ Grierson AJ. Review: the role of mitochondria in the pathogenesis of amyotrophic lateral sclerosis. Neuropathol Appl Neurobiol. (2011) 37:336–52. 10.1111/j.1365-2990.2011.01166.x 21299590

[7] Evans MC Couch Y Sibson N Turner MR. Inflammation and neurovascular changes in amyotrophic lateral sclerosis. Mol Cell Neurosci. (2013) 53:34–41. 10.1016/j.mcn.2012.10.008 23110760

[8] Philips T Robberecht W. Neuroinflammation in amyotrophic lateral sclerosis: role of glial activation in motor neuron disease. Lancet Neurol. (2011) 10:253–63. 10.1016/S1474-4422(11)70015-1 21349440

[9] Radford RA Morsch M Rayner SL Cole NJ Pountney DL Chung RS. The established and emerging roles of astrocytes and microglia in amyotrophic lateral sclerosis and frontotemporal dementia. Front Cell Neurosci. (2015) 9:414. 10.3389/fncel.2015.00414 26578880 PMC4621294

[10] Hall ED Oostveen JA Gurney ME. Relationship of microglial and astrocytic activation to disease onset and progression in a transgenic model of familial ALS. Glia (1998) 23:249–56. 10.1002/(Sici)1098-1136(199807)23:3<249::Aid-Glia7>3.0.Co;2-# 9633809

[11] Notturno F Capasso M DeLauretis A Carpo M Uncini A. Glial fibrillary acidic protein as a marker of axonal damage in chronic neuropathies. Muscle Nerve (2009) 40:50–4. 10.1002/mus.21323 19533665

[12] Notturno F Caporale CM De Lauretis A Uncini A. Glial fibrillary acidic protein: a marker of axonal Guillain-Barre syndrome and outcome. Muscle Nerve (2008) 38:899–903. 10.1002/mus.20983 18508349

[13] Su W Chen HB Li SH Wu DY. Correlational study of the serum levels of the glial fibrillary acidic protein and neurofilament proteins in Parkinson's disease patients. Clin Neurol Neurosurg. (2012) 114:372–5. 10.1016/j.clineuro.2011.11.002 22206859

[14] Katsanos AH Makris K Stefani D Koniari K Gialouri E Lelekis M. Plasma glial fibrillary acidic protein in the differential diagnosis of intracerebral hemorrhage. Stroke (2017) 48:2586–8. 10.1161/STROKEAHA.117.018409 28751552

[15] Norgren N Sundstrom P Svenningsson A Rosengren L Stigbrand T Gunnarsson M. Neurofilament and glial fibrillary acidic protein in multiple sclerosis. Neurology (2004) 63:1586–90. 15534240 10.1212/01.wnl.0000142988.49341.d1

[16] Yang Z Wang KK. Glial fibrillary acidic protein: from intermediate filament assembly and gliosis to neurobiomarker. Trends Neurosci. (2015) 38:364–74. 10.1016/j.tins.2015.04.003 25975510 PMC4559283

[17] Sofroniew MV Vinters HV. Astrocytes: biology and pathology. Acta Neuropathol. (2010) 119:7–35. 10.1007/s00401-009-0619-8 20012068 PMC2799634

[18] Rozanski M Waldschmidt C Kunz A Grittner U Ebinger M Wendt M. Glial fibrillary acidic protein for prehospital diagnosis of intracerebral hemorrhage. Cerebrovasc Dis. (2017) 43:76–81. 10.1159/000453460 27951536

[19] Ahadi R Khodagholi F Daneshi A Vafaei A Mafi AA Jorjani M. Diagnostic value of serum levels of GFAP, pNF-H, and NSE compared with clinical findings in severity assessment of human traumatic spinal cord injury. Spine (Phila Pa 1976) (2015) 40:E823–30. 10.1097/BRS.0000000000000654 25341992

[20] Benninger F Glat MJ Offen D Steiner I. Glial fibrillary acidic protein as a marker of astrocytic activation in the cerebrospinal fluid of patients with amyotrophic lateral sclerosis. J Clin Neurosci. (2016) 26:75–8. 10.1016/j.jocn.2015.10.008 26602604

[21] Yamanaka K Komine O. The multi-dimensional roles of astrocytes in ALS. Neurosci Res. (2018) 126:31–8. 10.1016/j.neures.2017.09.011 29054467

[22] Pehar M Vargas MR Cassina P Barbeito AG Beckman JS Barbeito L. Complexity of astrocyte-motor neuron interactions in amyotrophic lateral sclerosis. Neurodegener Dis. (2005) 2:139–46. 10.1159/000089619 16909019

[23] Clairembault T Kamphuis W Leclair-Visonneau L Rolli-Derkinderen M Coron E Neunlist M. Enteric GFAP expression and phosphorylation in Parkinson's disease. J Neurochem. (2014) 130:805–15. 10.1111/jnc.12742 24749759

[24] Van Dyke JM Smit-Oistad IM Macrander C Krakora D Meyer MG Suzuki M Macrophage-mediated inflammation and glial response in the skeletal muscle of a rat model of familial amyotrophic lateral sclerosis (ALS). Exp Neurol. (2016) 277:275–82. 10.1016/j.expneurol.2016.01.008 26775178 PMC4762214

[25] Suzuki M Tork C Shelley B McHugh J Wallace K Klein SM. Sexual dimorphism in disease onset and progression of a rat model of ALS. Amyotroph Lateral Scler. (2007) 8:20–5. 10.1080/17482960600982447 17364431

[26] Hayes-Punzo A Mulcrone P Meyer M McHugh J Svendsen CN Suzuki M Gonadectomy and dehydroepiandrosterone (DHEA) do not modulate disease progression in the G93A mutant SOD1 rat model of amyotrophic lateral sclerosis. Amyotroph Lateral Scler. (2012) 13:311–4. 10.3109/17482968.2012.654393 22409357 PMC3644484

[27] Howland DS Liu J She Y Goad B Maragakis NJ Kim B. Focal loss of the glutamate transporter EAAT2 in a transgenic rat model of SOD1 mutant-mediated amyotrophic lateral sclerosis (ALS). Proc Natl Acad Sci USA. (2002) 99:1604–9. 10.1073/pnas.032539299 11818550 PMC122237

[28] Nichols NL Gowing G Satriotomo I Nashold LJ Dale EA Suzuki M. Intermittent hypoxia and stem cell implants preserve breathing capacity in a rodent model of amyotrophic lateral sclerosis. Am J Respir Crit Care Med. (2013) 187:535–42. 10.1164/rccm.201206-1072OC 23220913 PMC3733409

[29] Turner MR. Progress and new frontiers in biomarkers for amyotrophic lateral sclerosis. Biomark Med. (2018) 12:693–6. 10.2217/bmm-2018-0149 29856233

[30] Jany PL Agosta GE Benko WS Eickhoff JC Keller SR Koehler W. CSF and blood levels of GFAP in Alexander disease. eNeuro (2015) 2:ENEURO.0080-15.2015. 10.1523/ENEURO.0080-15.2015 26478912 PMC4603256

[31] Mauer KR Lopatin RN Hoffman WA Grossman ET Russo RD. Decrease in a markedly elevated CA19-9 level after stenting of a benign pancreatic ductal stricture. Gastrointest Endosc. (1995) 42:261–3. 7498694 10.1016/s0016-5107(95)70103-6

